# Assessing Early Access to Care and Child Survival during a Health System Strengthening Intervention in Mali: A Repeated Cross Sectional Survey

**DOI:** 10.1371/journal.pone.0081304

**Published:** 2013-12-11

**Authors:** Ari D. Johnson, Dana R. Thomson, Sidney Atwood, Ian Alley, Jessica L. Beckerman, Ichiaka Koné, Djoumé Diakité, Hamed Diallo, Boubacar Traoré, Klenon Traoré, Paul E. Farmer, Megan Murray, Joia Mukherjee

**Affiliations:** 1 Malian Ministry of Health, Bamako, Mali; 2 Department of Global Health and Social Medicine, Harvard Medical School, Boston, Massachusetts, United States of America; 3 Department of Epidemiology, Harvard School of Public Health, Boston, Massachusetts, United States of America; 4 University of California San Francisco School of Medicine, San Francisco, California, United States of America; 5 Division of Global Health Equity, Brigham and Women’s Hospital, Boston, Massachusetts, United States of America; 6 Division of Research, Muso, Yirimadjo, Bamako, Mali; Tulane University School of Public Health and Tropical Medicine, United States of America

## Abstract

**Background:**

In 2012, 6.6 million children under age five died worldwide, most from diseases with known means of prevention and treatment. A delivery gap persists between well-validated methods for child survival and equitable, timely access to those methods. We measured early child health care access, morbidity, and mortality over the course of a health system strengthening model intervention in Yirimadjo, Mali. The intervention included Community Health Worker active case finding, user fee removal, infrastructure development, community mobilization, and prevention programming.

**Methods and Findings:**

We conducted four household surveys using a cluster-based, population-weighted sampling methodology at baseline and at 12, 24, and 36 months. We defined our outcomes as the percentage of children initiating an effective antimalarial within 24 hours of symptom onset, the percentage of children reported to be febrile within the previous two weeks, and the under-five child mortality rate. We compared prevalence of febrile illness and treatment using chi-square statistics, and estimated and compared under-five mortality rates using Cox proportional hazard regression. There was a statistically significant difference in under-five mortality between the 2008 and 2011 surveys; in 2011, the hazard of under-five mortality in the intervention area was one tenth that of baseline (HR 0.10, *p*<0.0001). After three years of the intervention, the prevalence of febrile illness among children under five was significantly lower, from 38.2% at baseline to 23.3% in 2011 (PR = 0.61, *p* = 0.0009). The percentage of children starting an effective antimalarial within 24 hours of symptom onset was nearly twice that reported at baseline (PR = 1.89, *p* = 0.0195).

**Conclusions:**

Community-based health systems strengthening may facilitate early access to prevention and care and may provide a means for improving child survival.

## Introduction

Despite substantial progress in reducing global child mortality over the past two decades [1], only 13 of 61 countries with high under-five mortality rates are currently on track to meet the fourth Millennium Development Goal: A 2/3 reduction in under-five child mortality by 2015 [2]–[4]. The leading contributors to child death in resource-limited settings are diarrheal disease, pneumonia, malaria, and neonatal illness [5], all diseases with well-validated and low-cost prevention methods and treatments [6], [7].

Yet these interventions are not reaching those who most need them [8] To address these disparities and accelerate progress on child survival, the Global Fund, the World Health Organization (WHO), the GAVI Alliance, the U.S. Global Health Initiative, and the World Bank have recently made health system strengthening a key priority for improving child survival and access to health care [9], [10].

To achieve access, utilization alone is insufficient. The Institute of Medicine (IOM) has defined health care access in terms of well-timed utilization: “the timely use of personal health services to achieve the best possible health outcomes” [11]. The World Health Organization has proposed an additional access measure that encompasses need, utilization, and quality of care termed “effective coverage”: the proportion of a population that needs a procedure that actually receives it” [12].

Previous studies document health system weaknesses that create barriers to timely utilization of prevention and care. Fees at point of care are associated with decreased and delayed utilization of care, and removing user fees correlates with increases in health care utilization, particularly among the poor [13]–[17]. Geographic barriers, poverty, lack of infrastructure, and inadequate staffing also impede timely access [18].

Timely access is particularly important for leading causes of child mortality, which progress quickly. Poor patients are also more likely to access care late in the course of their illnesses [18]. Delayed access to effective care has been correlated with increased under-five mortality from malaria, neonatal asphyxia, acute respiratory infection, and diarrheal disease [19]–[26].

We implemented a health system strengthening intervention in peri-urban Mali designed to improve child survival by improving rapid access to prevention and treatment. The intervention focused on removing access barriers though Community Health Worker (CHW) active case finding, the removal of user fees for the poor, strengthened clinical infrastructure, a rapid referral network to link community members to the health system, and a package of prevention services addressing conditions of poverty. We used the IOM and WHO definitions of access: the proportion of a population that needs a health intervention and actually receives it, within the time-frame necessary to achieve optimal health outcomes [11], [12]. Here, we describe the implementation of this intervention and document the changes in under-five mortality and early malaria treatment that occurred in the intervention area over a three year period from 2008 to 2011.

## Methods

### Ethics Statement

This study was reviewed and approved by the University of California San Francisco Human Research Protection Program Committee on Human Research, IRB #10-02198, Reference #004193. All respondents provided written informed consent. Written informed consent was obtained from the parents/guardians of respondents younger than 18 years of age.

### Study Setting

The non-governmental organization Muso and the Malian Ministry of Health (MoH) launched their health system strengthening intervention in the periurban area of Yirimadjo in Mali in September 2008. Mali is ranked amongst the world’s poorest countries and has the world’s eighth highest under-five child mortality rate, projected to be 128/1000 live births in 2012 [27]–[28]. Since the Bamako Initiative of 1987 [29], the public sector health system has utilized a user fee for service health care financing model. Approximately 3.5 square miles on the outskirts of Bamako, Yirimadjo has an estimated population of 56,000, and has experienced rapid population growth due to high birth rates and in-migration. At baseline, the public sector health provided primary care through a primary care center that consisted of a consultation room, medicine dispensary, observation room, and two rooms for labor and delivery. Relays, local volunteers, had been trained through the MoH to share key messages with other community members about maternal-child health. At baseline, there were no Community Health Workers providing community-based management of childhood illness.

### Procedures and Description of the Health System Strengthening Intervention

In partnership with local, regional, and national structures of the Malian MoH, Muso undertook a three-component intervention to overcome health system barriers to timely access to preventative and curative care (Table 1). The model is diagrammed in Figure S1.

**Table 1:** Muso’s Health System Strengthening Model.

Intervention	Core Strategies
Mobilizing the health care delivery system	Active door-to-door case finding by Community Health Workers, who proactively identified children with 16 danger signs of childhood illness, diagnosed and treated pediatric malaria in the home, referred and accompanied other cases to the health center, conducted follow-up visits at 24 and 48 hours, and connected pregnant women with prenatal care and facility-based delivery
	Removing user fees to provide free access to care to all patients who could not afford to pay
	Constructing and renovating clinical infrastructure
Creating rapid referral networks	Community organizers, religious leaders, educators mobilize community members to bring children in early for prevention and care services
Overcoming conditions of poverty	Providing a package of programs to prevent childhood illness by addressing conditions of poverty, through non-formal education, microenterprise, and community organizing

The first component of the intervention involved (i) selecting, training, and employing Community Health Workers to conduct active door-to-door case finding, identifying 16 danger signs and symptoms of childhood illness for children 0–59 months, diagnosing malaria in the home using HRP-2 rapid antigen diagnostic testing, treating malaria in the home with artemisinin-based combination therapy (ACT), referring or accompanying patients with other illnesses to a MoH community health center, conducting follow-up visits for treated patients at 24 and 48 hours, and connecting pregnant women with prenatal care and birthing services (ii) removing user fees to provide free care for all patients who could not afford to pay, as determined by patient self-reporting to their CHW that they were unable to pay (iii) constructing and renovating clinical infrastructure at the MoH community health center and (iv) training of health care providers at the MoH community health center. User fee removal, CHWs, and health center construction were all fully deployed by September 2008.

A team of 20 CHWs and 3 CHW assistants provided outreach and care to the 11,000 households in the area of the intervention. Residents of the communities they serve, this new cadre of community-based health professionals were nominated by their communities and selected by a committee that included representatives of the community, the MoH health center, and Muso. The CHWs were jointly trained and supervised by Muso and the MoH health center clinical team. The area of the intervention, estimated population 56,000, was divided into 20 zones, each served by a CHW. Thus each CHW provided outreach and care to a zone with an average population of 2800, of which 560 were children aged 0–59 months. CHW diagnosis and treatment algorithms were built from WHO recommendations for the Integrated Management of Childhood Illness. These CHW protocols (Figure S2 and Figure S3) are included as supporting information.

The second component of the intervention involved the creation of a rapid referral network to identify sick children and refer them early for assessment. The rapid referral network included 13 local religious leaders, 14 education centers, and 238 community organizers, as well as the households they engaged through outreach work. Members of this network trained in the importance of early diagnosis and treatment of sick children. They identified patients, particularly children, who were sick and referred them to a CHW for assessment. They also taught their neighbors and family members about the importance of early diagnosis and treatment for children with fever.

The third component addressed the socioeconomic determinants of health disparities through microenterprise support, education, and community organizing. A microenterprise program provided training, savings structures, and low-interest and no-interest loans to women to start or expand revenue generating activities. The microenterprise program was designed to increase women’s income. In doing so, the program aimed to increase women’s decision-making power relative to health decisions for themselves and their children, as well as to increase their capacity to spend on health related commodities, such as soap and nutritious foods. Testing these specific hypotheses was beyond the scope of the current study and will be the subject of future research. The non-formal education curriculum, developed and implemented by the organization Tostan, is a human rights-based curriculum offered to adults and adolescents, which trains community members in community-led development, improving living conditions that influence community health. The three-year curriculum includes modules on human rights, problem solving, project management, democracy, health, hygiene, literacy, numeracy and income generation skills. Health skills modules include family planning, prenatal and perinatal health, vaccinations, malaria prevention and care, diarrheal disease prevention and home-based management, and nutrition. Classes met eight hours per week during the three year curriculum. Muso and Tostan together trained 238 Community Organizers to identify and implement community-designed health and development projects.

### Participants and Sampling Methodology

We conducted independent, randomized cross-sectional household surveys at baseline, 12, 24, and 36 months in the area of intervention starting in June 2008. We included households with a woman aged 16 and older. We sampled 400 households in June 2008 (baseline), June 2009 (12 months), June 2010 (24 months), and 1170 households in June 2011 (36 months). We powered the sample to estimate the percentage of under-five children with a fever in the two weeks prior who received an effective antimalarial within 24 hours of symptom onset, in a population of 56,000, with a 95% confidence level, a 5% margin of error, and a conservative 50% response distribution.

We utilized a two-stage cluster sampling methodology with probability of selection proportionate to size to achieve a self-weighting sample of households. In the first-stage of sampling, we generated 40 (2008, 2009, 2010) or 65 (2011) random non-overlapping latitude-longitude coordinates with 100 m radial buffers within the intervention area. Using less than six month old satellite imagery, we counted the number of structures within each cluster as a measure of population density. In the second stage of sampling, interviewers selected households by arriving at the center of the cluster, spinning a pen to identify a random direction, and visiting every second household and choosing a pre-determined number of households proportional to the population density in that cluster. Within each household, one respondent, a female aged 16 or over, was randomly selected from all eligible respondents based on a KISH table. The survey was adapted from existing validated tools created by ICF International [30] and Population Services International (PSI) and included questions about household demographics, fever in children under five, and birth histories (10-year birth histories in 2008, and 6-year birth histories in 2009–2011). Interviewers were hired for the sole purpose of survey administration, and were not members of the communities they surveyed.

### Patient Visit Data and Program Outputs

Data on patient encounters were collected through patient encounter forms completed by MoH Primary Health Center staff and CHWs. The number of patient encounters in which user fees were effectively removed was tracked through an electronic database maintained by a data entry professional based at the Primary Health Center. The number of sick patient visits per month was tracked by the Primary Health Center and provided through the Ministry of Health District Health Information Systems office. Program staff submitted forms to track other program output including education center enrollment and microloan repayment rates, which were then verified by program supervisors.

To track exposure to Community Health Worker outreach, we added questions to the 2011 household survey regarding whether and how recently each household had been visited by a CHW.

### Analysis

We conducted an adequacy evaluation based on a full coverage intervention to measure changes in early access and under-five mortality outcomes in the surveyed area [31].

### Under-five Mortality

We calculated under-five mortality rates from all births reported by respondents in the five years prior to the survey. We calculated under-five mortality rates and compared risk of death before age five across surveys with Cox proportional hazards regression using survey year as the explanatory variable. Children still alive and under age five at the time of survey were right censored. We accounted for clustering of observations by sampling area with the shared frailty option and compared the difference in under-five mortality rates across surveys with the 2008 survey as the reference. The proportional hazard criteria were met. We multiply imputed missing age of death, month of birth, and year of birth based on other covariates using the MICE system of chained equations, and analyzed the results in Stata (v.10) using the multiply imputed datasets to preserve the original sample variability including variability due to missing data.

### Analysis of Fever and Early Effective Antimalarial Treatment

We calculated the proportion of children in the household under age five (both biological and non-biological children of respondents) who were reported to be febrile in the prior two weeks and the proportion of febrile children under-five who received an effective antimalarial treatment within 24 hours of symptom onset. We estimated prevalence in SAS (v.9.2) accounting for clustering with the surveyfreq command, and compared estimates with chi-square statistics, reporting prevalence ratios and p-values with 2008 as the reference.

## Results

Demographic characteristics of the female respondents showed that there was little change in the average age, marital status, or household size of respondents over the four years of surveys. Fewer respondents attended school in 2009 and 2010 compared to 2008 (*p*<0.05), and among school attendees, respondents completed fewer grades in the surveys after 2008 (*p*<0.05) (Table 2).

**Table 2:** Respondent Demographic and Socioeconomic Characteristics, 2008–2011.

Variable	Year	N	Missing observations	Summary statistic	p-value of difference from baseline†	p-value of overall difference†
Median age	2008	386	0	29 (25, 36)	ref	0.0025
	2009	394	0	28 (23, 35)	0.2516	
	2010	407	0	28 (23, 33)	0.0001	
	2011	1040	0	28 (23, 35)	0.0299	
Percent women who are married	2008	386	3	90.6	ref	0.1438
	2009	394	0	90.1	0.8138	
	2010	407	1	93.4	0.1545	
	2011	1040	1	89.4	0.4957	
Percent women who attended any school	2008	386	0	51.0	ref	0.0014
	2009	394	0	38.6	0.0005	
	2010	407	0	41.5	0.0072	
	2011	1040	0	46.8	0.1455	
Of women who attended anyschool, median level completed(1–17 grades)	2008	197	0	8 (5, 9)	ref	0.0003
	2009	152	0	6 (4, 9)	0.0403	
	2010	169	0	6 (4, 8)	<0.0001	
	2011	533	0	6 (4, 9)	0.0002	
Median household size	2008	386	208	5 (3, 6)	ref	0.0824
	2009	394	0	5 (4, 7)	0.6066	
	2010	407	0	5 (4, 6)	0.1708	
	2011	1040	3	5 (4, 7)	0.7862	

† median, 25% and 75% percentiles, and *F*-test reported for continuous variables; percent and *X^2^*-test reported for categorical variables.

In the 2011 household survey, 54% of respondents reported they had been visited by a CHW in the prior 3 years. Of those who had been visited by a CHW, 60% reported a visit within the past month, 78% reported a visit within the past three months (Tables S1 and S2).

### Intervention Outputs

During the period of the intervention, CHWs performed on average 7,109 screening home visits per month actively searching for cases of sick children. CHWs identified sick children at an average of 356 sick child assessments per month, 12,120 sick child home visits between 2008 and 2011. They performed malaria rapid antigen diagnostic testing at 6,943 of those visits, an average of 204 malaria rapid diagnostic tests per month. CHWs identified one or more danger signs of severe malaria, pneumonia, diarrheal disease, or other severe childhood illness requiring immediate accompaniment for health center level care during 4,925 of those home visits. Of the sick children they cared for, they reached 35% within 24 hours of symptom onset, 52% within 48 hours of symptom onset, and 78% within 72 hours of symptom onset (Table 3).

**Table 3:** Key Intervention Outputs, September 2008 to June 2011.

Community Health Worker active case finding home visits per month	7,109
CHW active case finding home visits per household per month (for an estimated 11,000 households)	0.65
CHW sick child assessments per month	356
CHW malaria rapid antigen diagnostic tests in the home per month	204
CHW identification of cases with danger signs requiring immediate referral, per month	145
Percentage of CHW patients 0–59 months assessed within 24 hours of symptom onset	35%
Percentage of CHW patients 0–59 months assessed within 48 hours of symptom onset	52%
Percentage of CHW patients 0–59 months assessed within 72 hours of symptom onset	78%
Total number of sick patient visits at the MoH health center during the period of the intervention, September 2008 to June 2011	66,631
Number of sick patient clinic visits per person (based on estimated population of 56,000) at the MoH health center duringthe period of the intervention	1.19
Total number of sick patient visits with user fees removed at MOH health center, September 2008 to June 2011	33,800

**Figure 1:**
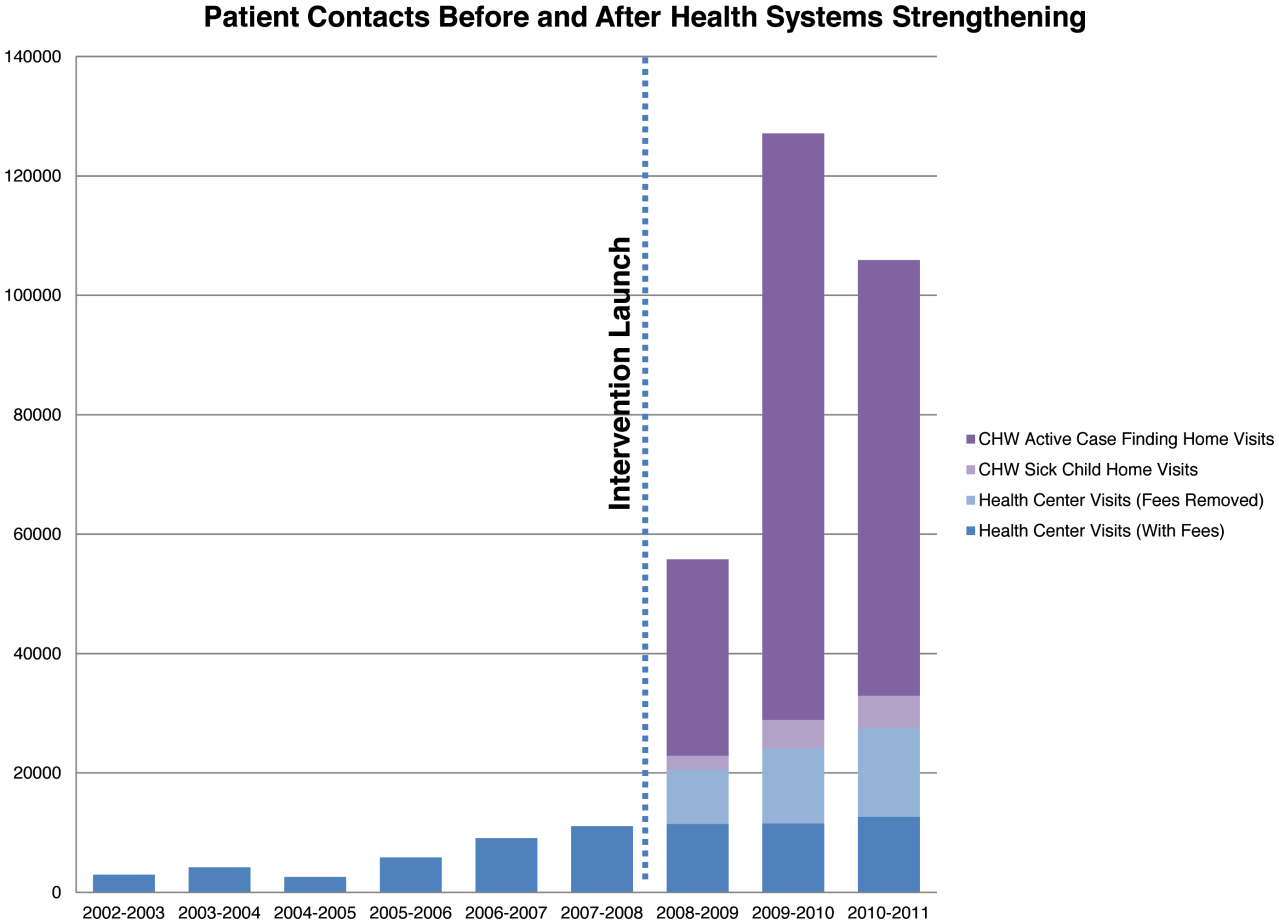
Patient Visits Before and After the Launch of the Health System Strengthening Intervention.

A 2900 square foot clinical care center was constructed for the MoH Yirimadjo Community Health Center and opened September 2008. The expanded Yirimadjo Community Health Center provided 66,631 sick patient consultations during the period of the intervention (September 2008 to June 2011). User fees were removed for consultations, diagnostic studies, services, and medications at 33,800 of those health center visits, for those patients who were determined by CHWs to be unable to pay for care themselves. Figure 1 depicts patient visits at the Health Center and in the community from 2002 to 2011, before and after the September 2008 launch.

Community Organizers implemented community initiatives including a potable water advocacy campaign, after which the government installed more than 30 new clean water access points in the area. The microenterprise program provided training loans to 247 women, who achieved a greater than 99% repayment rate. 1245 adults and adolescents participated in the non-formal education program across 14 education centers.

### Child Mortality

**Figure 2:**
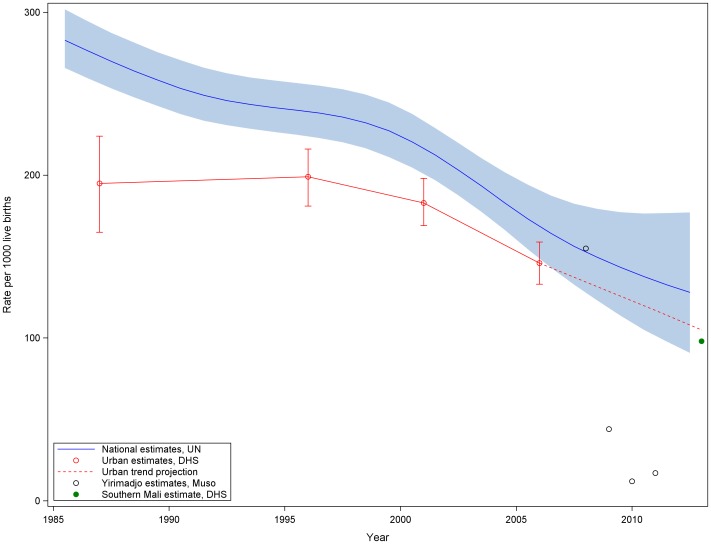
Under-Five Mortality in Muso Catchment Area Compared to Trends in Under-Five Mortality in Urban Areas and Nationally.

Figure 2 shows the downward mean trend in under-five mortality over the four surveys, with under-five mortality starting at a pre-intervention rate of 155 deaths per 1000 live births, and ending at 17 deaths per 1000 live births in 2011. We present these results against the trends of annual Demographic and Health Survey (DHS) urban under-five mortality estimates and annual UN national estimates of under-five mortality derived from DHS and census data (Figure 2). In the first year of the intervention, hazard of death before age five were nearly quartered (Hazard Ratio (HR) = 0.27, p = 0.0020). In 2010 and 2011, the hazards of under-five mortality were approximately one tenth that of baseline (2010 HR = 0.07, 2011 HR = 0.10, *p*<0.0001 for both ratios) (Table 4).

**Table 4:** Under Five Mortality Rate in Muso Catchment Area, 2008–2011.

Year	Number of births last 5 years (raw)	Number ofdeaths (raw)	Number of observations imputed due to missingbirth or death data	Average number of birthsin the last 5 years (N)used in imputations	Average numberof deaths used in imputations	Under five mortality rate (per 1000 live births)	Hazard Ratio(versus 2008)	p-value
2008	214	26	196	316	38	155	1.00	Ref
2009	394	19	73	443	21	44	0.27	0.0020
2010	509	8	4	511	8	12	0.07	<0.0001
2011	1144	22	317	1390	29	17	0.10	<0.0001

### Early Treatment Initiation, Prevalence of Febrile Illness

The prevalence of febrile illness in the two weeks prior to the survey among children under-five was 38.2% in 2008, and 23.3% in 2011 (Prevalence Ratio (PR) = 0.61, *p* = 0.0009) (Table 5). Among febrile children, the proportion that received antimalarial treatment within 24 hours of symptom onset nearly doubled during the study period (PR = 1.89, *p* = 0.0195) (Table 6).

**Table 5:** Febrile Illness in Children Under Five within Muso Catchment Area, 2008–2011.

Outcome	Year	N	Percent	Prevalence Ratio (versus 2008)	p-value
Fever in children under five in the last two weeks	2008	301	38.2	1.00	Ref
	2009	431	21.8	0.57	0.0006
	2010	484	25.4	0.67	0.0196
	2011	1288	23.3	0.61	0.0009

**Table 6:** Timely Effective Antimalarial Treatment within 24 Hours (Among Children Under Five with Fever) within the Muso Catchment Area, 2008–2011.

Outcome	Year	N	Percent	Prevalence Ratio (versus 2008)	p-value
Among children under five with a fever in the lasttwo weeks, children who received effectiveantimalarial treatment within 24 hours	2008	115	14.8	1.00	Ref
	2009	94	29.8	2.01	0.0116
	2010	123	44.7	3.02	<0.0001
	2011	300	28.0	1.89	0.0195

## Discussion

Timely access to prevention resources and early treatment can prevent death and interrupt transmission, thus reducing the overall burden of childhood illnesses and under-five child mortality. Yet it is not clear what package of health system improvements can best achieve these goals. The health system strengthening intervention described here integrated several key strategies to achieve early access to prevention and care: Active case finding by CHWs to identify sick children earlier in the course of their illness, a rapid referral network to mobilize community members to bring sick children in for care earlier, user fee removal to eliminate financial barriers that delay care, and programs to address the socioeconomic determinants of health. In anticipation of a marked increase in health services utilization through these strategies, the intervention built MoH primary health center capacity through new construction, renovation, equipment, and training. Strong partnership with local, regional, and national structures of the Malian Ministry of Health aligned the intervention’s strategies and aims with national objectives.

During the health system strengthening intervention described here, the rate of early treatment with an effective antimalarial doubled. Twenty-four hour initiation of malaria treatment peaked in year two of the intervention (PR = 3.02). We can hypothesize two possibilities for why the early treatment rate was higher in year two than in year three: First, while the population continued to grow, the number of CHWs serving that population remained constant. Second, the catchment area of 560 children per CHW may have exceeded their capacity for sustained outreach. In year two of the intervention, CHWs were frequently working overtime hours to cover their large catchment areas, and could not sustain this during year three of the intervention. In either scenario, an increase in the ratio of CHWs-to-population may have facilitated further gains in early treatment.

The percentage of children with febrile illness in the study area was significantly different over the three years of the intervention, an absolute decrease of 15% and a relative decrease of 39% between estimates. A reduced burden of childhood illness could have resulted from increased rapid treatment and increased access to prevention resources such as potable water, both of which would reduce the prevalence of childhood febrile illnesses.

During this same period from 2008 to 2011, we also documented a significant difference in under-five child mortality in the study area. A stronger health system may have created the conditions for a decline in mortality: Early access to effective treatment, programming to improve the socioeconomic determinants of disease, increased access to prevention resources, and decreased prevalence of childhood febrile illness could have each contributed to this rapid decrease in child mortality.

While these improvements are impressive, the study has several important limitations. First, the sample size of 400 households for the first three surveys was powered for the outcome of early effective malaria treatment. The sample size was not powered for the measurement of under-five child mortality rates. We nonetheless observed a significant downtrend in under-five child mortality after the start of the intervention with respect to baseline, which we report. Second, the study does not permit causal association between intervention and outcomes. Without a control arm, we were unable to determine if the differences observed over the course of the study were due to the intervention, or to demographic shifts in the study population, general national trends in health, or other factors. Third, we collected only the last six years of birth histories which limited the amount of data that could be included in the child mortality analysis, and could have led to underreporting of child deaths that occurred close to six years before the survey. Further, the study did not collect information regarding household wealth, parasite prevalence, or vaccination coverage. The demographic characteristics measured in this study did not provide evidence for an improving risk factor profile of those surveyed, as respondents in later surveys were younger and less-educated than respondents at baseline (Table 2).

Ideally we would compare outcomes in the intervention area with similar areas that did not have the intervention. We planned to use repeated cross sectional surveys to compare the impact of the intervention against similar figures for Mali nationally and in all other urban areas, as measured by the DHS of 2006 and the DHS planned for 2011. Unfortunately the scheduled 2011 DHS was postponed due to conflict and when completed in 2012–2013 it excluded the northern half of the country. At the time of this writing, preliminary 2012–2013 DHS results have been released, but final datasets are not yet published. Therefore, we compare our results to two projections (Tables S3 and S4) and to preliminary results from the partial DHS [28], [32]. First, the United Nations Inter-agency Group for Child Mortality Estimation projected that Mali’s national annual under-five mortality rate would decrease from 150/1,000 in 2008 to 133/1,000 in 2011. Second, using 2006 Mali DHS data from urban households, we project an under-five mortality rate of 117/1000 in urban Mali in 2011 by extending the percent decline in child mortality between 2001 and 2006. The 2009–2013 under-five mortality rate for southern Mali reported in the preliminary DHS results was 98/1000. We measured under-five mortality in the area of the intervention to be 17/1000 in 2011 (Figure 2).

While a two-stage cluster design was used, based on satellite images that were less than six months old, data were not weighted to account for discrepancies between estimated cluster size (from the satellite imaging sampling frame) and actual clusters sizes of those selected from complete enumeration of households in the field, which is the gold standard.

The study did not measure the proportion of sick children who were referred through the rapid referral network, identified through Community Health Worker active case finding, or brought independently to the community health center by their family. We also did not measure the proportion of sick children in the survey for whom user fees were removed. We therefore cannot quantify the relative contributions of CHW active case finding, community mobilization through the rapid referral network, user fee removal, and health center based systems strengthening in facilitating early access, reduced under-five morbidity and reduced mortality.

The study did not measure sufficient intermediate changes to elucidate the role of the microenterprise and education components of the intervention in improving access or child health outcomes. The study was also not designed to assess the sensitivity or specificity of the user fee exemption strategy employed, nor its impact on equity in health access. Further controlled studies are needed to assess the relative contributions of each element of the intervention to improving early access and child survival.

High rates of under-five child mortality are due in part to health system weakness: the inability to equitably and rapidly deliver evidence-based child survival tools. The study’s findings build upon and are consistent with prior research in Mali: CHW contact was associated with increased health knowledge [33], [34], and a combination of CHW deployment and user fee removal was associated with increased utilization and reduced child mortality [35], [36]. Recent studies in Ghana and Egypt have also supported the potential of health systems strengthening interventions to reduce child mortality [37], [38]. To strengthen health system capacity to reach children quickly, the model tested here employed a proactive approach, actively seeking out patients door-to-door in the catchment area, moving first point of contact into the home, and mobilizing community networks to engage in prevention and care. The current study shows a rapid increase in early access to care and a rapid lowering of child morbidity and mortality in the area of this health systems strengthening intervention. Health system strengthening, by overcoming a common set of barriers to timely access, can potentiate investments across multiple disease areas and catalyze global improvements in health and survival.

## Supporting Information

Figure S1 **Health Systems Strengthening Model for Early Access and Improved Child Survival.**
(TIF)

Figure S2 **Protocol: CHW Encounter with Sick Child.**
(TIF)

Figure S3 **Protocol: CHW Encounter with Pregnant Woman.**
(TIF)

Table S1 **Households Reporting a Visit by a CHW in the Previous 3 Years (2011 Survey).**
(DOCX)

Table S2 **Among Households Who Reported Having Received a CHW Home Visit, Timing of the Most Recent CHW Home Visit (2011 Survey).**
(DOCX)

Table S3 **Annual Under-Five Mortality Rate (Urban residents only), DHS (1987–2006).**
(DOCX)

Table S4 **Annual Under-Five Mortality Rate, National Mid-Year Estimates and Projections, UN Inter-agency Group for Child Mortality Estimation (1980–2012).**
(DOCX)
