# Supplementary figures and images for: Assessing Early Access to Care and Child Survival during a Health System Strengthening Intervention in Mali: A Repeated Cross Sectional Survey
Source: PLoS One. 2013 Dec 11;8(12):e81304. doi: 10.1371/journal.pone.0081304 (PMC3859507; doi:10.1371/journal.pone.0081304)

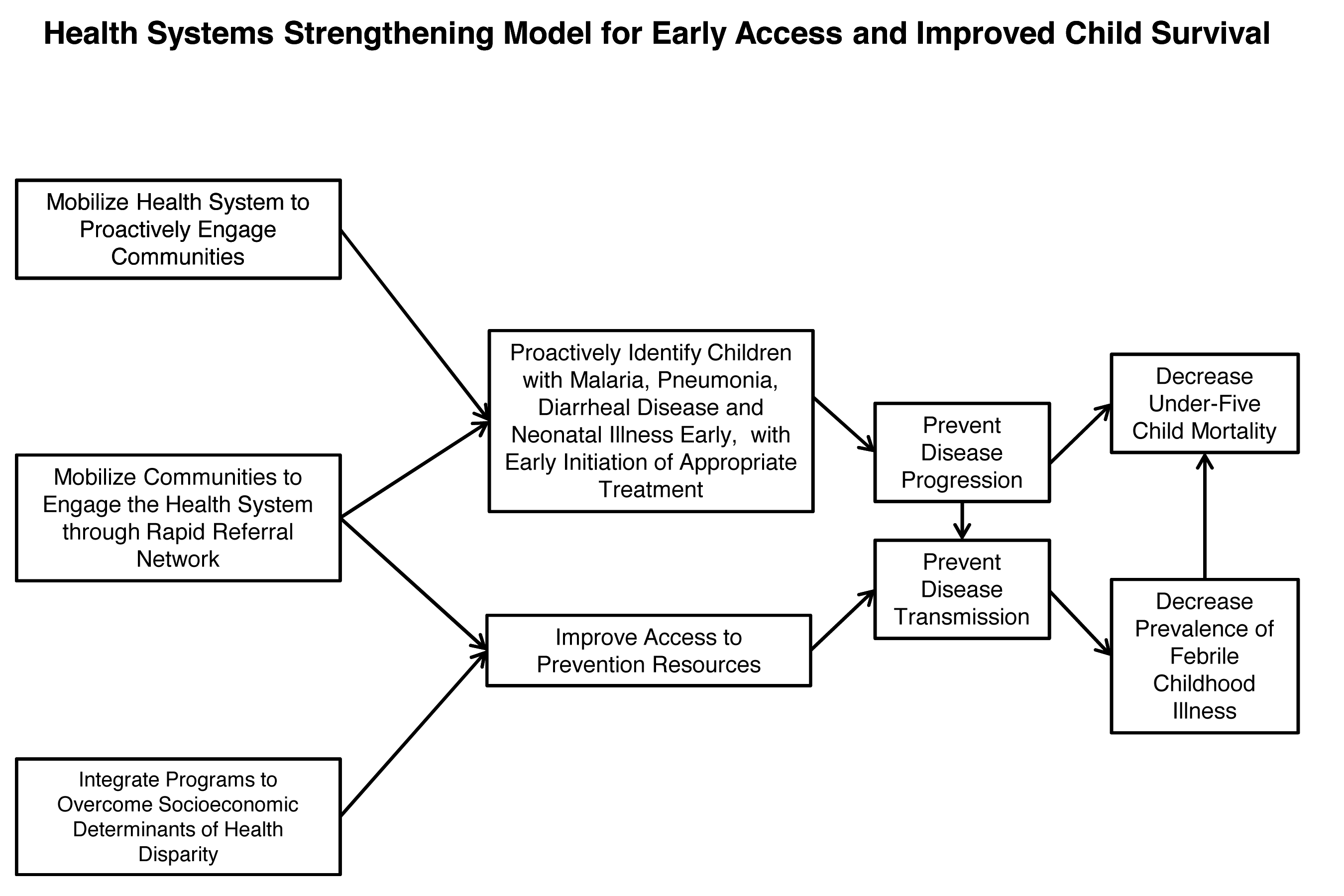

Supplement: Figure S1 — Health Systems Strengthening Model for Early Access and Improved Child Survival. (TIF) [file pone.0081304.s001.tif]

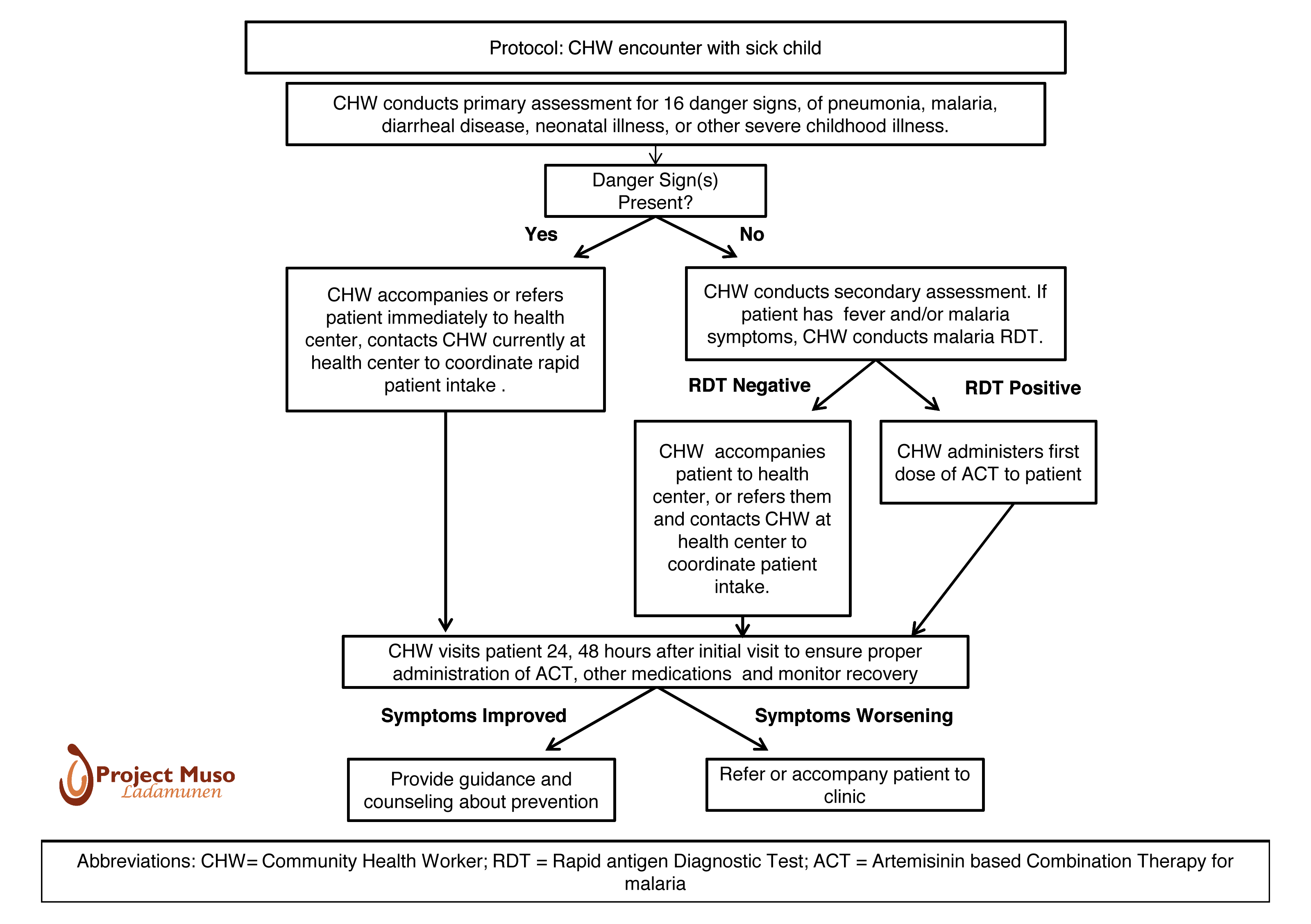

Supplement: Figure S2 — Protocol: CHW Encounter with Sick Child. (TIF) [file pone.0081304.s002.tif]

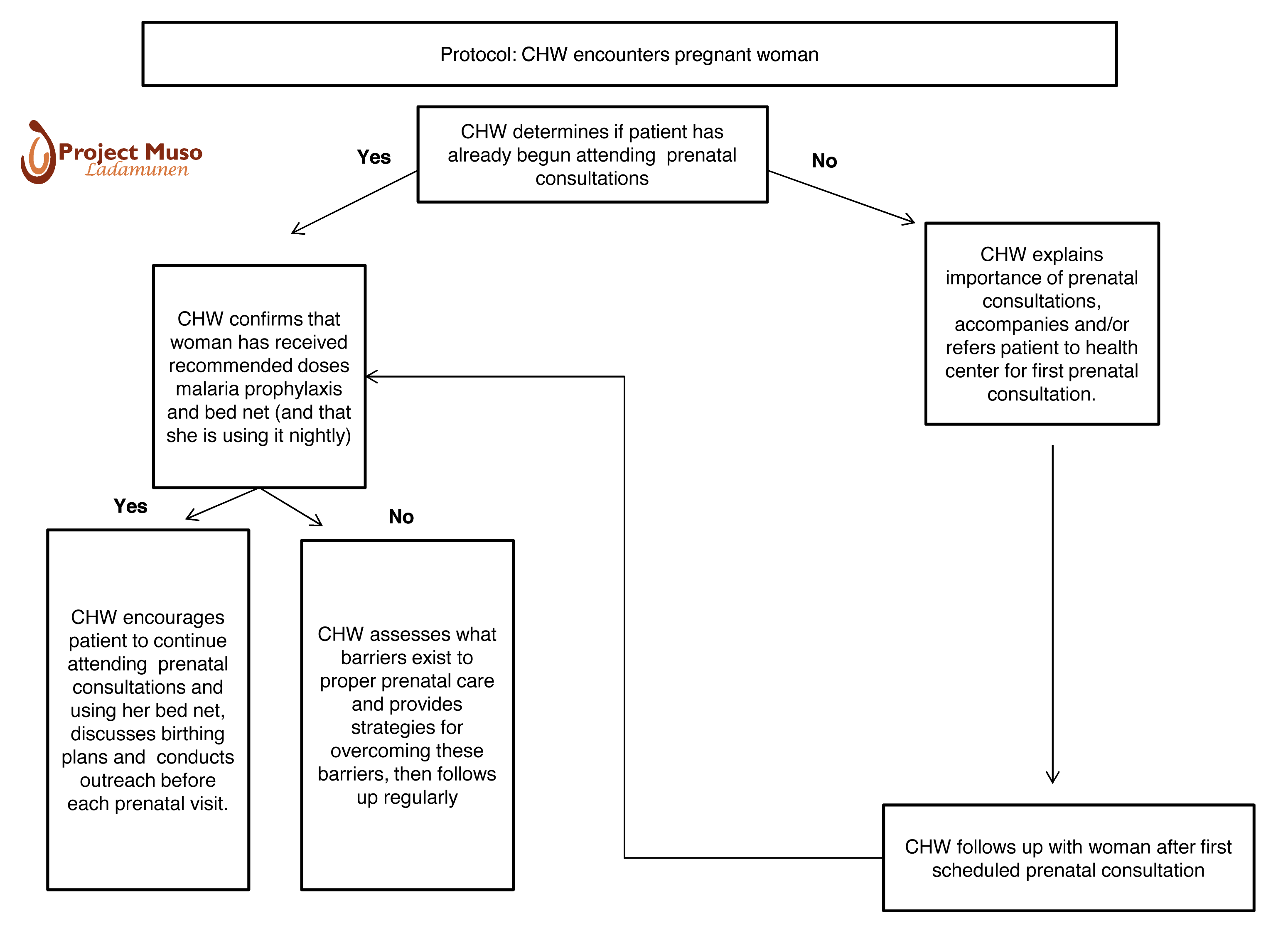

Supplement: Figure S3 — Protocol: CHW Encounter with Pregnant Woman. (TIF) [file pone.0081304.s003.tif]
