# Supplementary material for: Assessing Early Access to Care and Child Survival during a Health System Strengthening Intervention in Mali: A Repeated Cross Sectional Survey
Source: PLoS One. 2013 Dec 11;8(12):e81304. doi: 10.1371/journal.pone.0081304 (PMC3859507; doi:10.1371/journal.pone.0081304)
Supplement: Table S1 — Households Reporting a Visit by a CHW in the Previous 3 Years (2011 Survey). (DOCX) [file pone.0081304.s004.docx]

**Table S1. Households Reporting a Visit by a CHW in the Previous 3 Years (2011 Survey)**

|  | **Frequency** | **Percent** | **Cumulative Frequency** | **Cumulative Percent** |
| --- | --- | --- | --- | --- |
| *Yes* | 632 | 54.16 | 632 | 54.16 |
| *No* | 522 | 44.73 | 1154 | 98.89 |
| *No response* | 13 | 1.11 | 1167 | 100.00 |
