# Supplementary material for: Assessing Early Access to Care and Child Survival during a Health System Strengthening Intervention in Mali: A Repeated Cross Sectional Survey
Source: PLoS One. 2013 Dec 11;8(12):e81304. doi: 10.1371/journal.pone.0081304 (PMC3859507; doi:10.1371/journal.pone.0081304)
Supplement: Table S2 — Among Households Who Reported Having Received a CHW Home Visit, Timing of the Most Recent CHW Home Visit (2011 Survey). (DOCX) [file pone.0081304.s005.docx]

|  | **Frequency** | **Percent** | **Cumulative Frequency** | **Cumulative Percent** |
| --- | --- | --- | --- | --- |
| One week | 212 | 33.54 | 212 | 33.54 |
| One month | 166 | 26.27 | 378 | 59.81 |
| Three months | 115 | 18.20 | 493 | 78.01 |
| One Year | 65 | 10.28 | 558 | 88.29 |
| More than a year | 35 | 5.54 | 593 | 93.83 |
| Do not know | 39 | 6.17 | 632 | 100.00 |

**Table S2. Among Households Who Reported Having Received a CHW Home Visit, Timing of the Most Recent CHW Home Visit (2011 Survey)**
