# Supplementary material for: Assessing Early Access to Care and Child Survival during a Health System Strengthening Intervention in Mali: A Repeated Cross Sectional Survey
Source: PLoS One. 2013 Dec 11;8(12):e81304. doi: 10.1371/journal.pone.0081304 (PMC3859507; doi:10.1371/journal.pone.0081304)
Supplement: Table S3 — Annual Under-Five Mortality Rate (Urban residents only), DHS (1987–2006). (DOCX) [file pone.0081304.s006.docx]

**Table S3.** **Annual Under-Five Mortality Rate (Urban residents only), DHS (1987-2006)**

| **Year** | **Risk of dying by age 5 per 1000 live births in the one year before survey (95% CI)** |
| --- | --- |
| **1987** | **195 (165, 224)** |
| **1996** | **199 (181, 216)** |
| **2001** | **183 (169, 198)** |
| **2006** | **146 (133, 159)** |
