# Supplementary material for: Assessing Early Access to Care and Child Survival during a Health System Strengthening Intervention in Mali: A Repeated Cross Sectional Survey
Source: PLoS One. 2013 Dec 11;8(12):e81304. doi: 10.1371/journal.pone.0081304 (PMC3859507; doi:10.1371/journal.pone.0081304)
Supplement: Table S4 — Annual Under-Five Mortality Rate, National Mid-Year Estimates and Projections, UN Inter-agency Group for Child Mortality Estimation (1980–2012). (DOCX) [file pone.0081304.s007.docx]

**Table S4. Annual Under-Five Mortality Rate, National Mid-Year Estimates and Projections, UN Inter-agency Group for Child Mortality Estimation (1980-2012)**

| **Year** | **Mean** | **Minimum** | **Maximum** |
| --- | --- | --- | --- |
| 1980.5 | 320.9 | 300.4 | 343.2 |
| 1981.5 | 313.2 | 293.8 | 334.9 |
| 1982.5 | 305.4 | 286.7 | 326.3 |
| 1983.5 | 297.8 | 279.4 | 318.1 |
| 1984.5 | 290 | 272.5 | 309.8 |
| 1985.5 | 282.9 | 265.8 | 301.8 |
| 1986.5 | 276.3 | 259.4 | 294.5 |
| 1987.5 | 269.9 | 253.3 | 287.4 |
| 1988.5 | 263.9 | 247.7 | 281.4 |
| 1989.5 | 258.5 | 242.5 | 275.5 |
| 1990.5 | 253.3 | 237.5 | 270.6 |
| 1991.5 | 249 | 233.4 | 265.9 |
| 1992.5 | 245.8 | 230.7 | 262.5 |
| 1993.5 | 243.4 | 228.5 | 259.9 |
| 1994.5 | 241.5 | 226.6 | 258.2 |
| 1995.5 | 239.9 | 224.8 | 256.6 |
| 1996.5 | 238.1 | 222.7 | 254.9 |
| 1997.5 | 235.7 | 220.1 | 252.7 |
| 1998.5 | 232.1 | 216.5 | 249.5 |
| 1999.5 | 227.2 | 211 | 244.6 |
| 2000.5 | 220.4 | 204.6 | 237.6 |
| 2001.5 | 212.4 | 196.8 | 228.9 |
| 2002.5 | 203.1 | 187.6 | 219.7 |
| 2003.5 | 193.3 | 177.4 | 210.3 |
| 2004.5 | 183 | 166.3 | 201.7 |
| 2005.5 | 173.1 | 154.5 | 194.1 |
| 2006.5 | 164.2 | 143.1 | 187.4 |
| 2007.5 | 156.2 | 132.6 | 182.4 |
| 2008.5 | 149.5 | 122.9 | 179.2 |
| 2009.5 | 143.4 | 113.7 | 177.2 |
| 2010.5 | 137.8 | 105.1 | 176.4 |
| 2011.5 | 132.7 | 97.8 | 176.6 |
| 2012.5 | 128 | 90.8 | 177.1 |

Source: UN Inter-agency Group for Child Mortality Estimation
